# Comparison of the Quality of Life of Physically Active and Inactive Grandmothers Caring and Non-caring for Grandchildren: A Cross-Sectional Study

**DOI:** 10.4314/ejhs.v33i2.14

**Published:** 2023-03

**Authors:** Homa Hemmati, Ali Golestani, Mitra Hashemi, Mahbubeh Tabatabaeichehr, Hamed Mortazavi

**Affiliations:** 1 Geriatric Care Research Center, Department of Geriatric Nursing, School of Nursing, North Khorasan University of Medical Sciences, Bojnurd, Iran; 2 Department of Sport Sciences, University of Bojnurd, Bojnurd, Iran; 3 North Khorasan University of Medical Sciences, Bojnurd, Iran; 4 Department of Midwifery, School of Medicine, Gerontological Care Research Center, North Khorasan University of Medical Sciences, Bojnurd, Iran

**Keywords:** Quality of Life, Grandparents, Child Care, Sedentary Behavior

## Abstract

**Background:**

Nowadays, grandparents have a major role in taking care of their grandchildren. Also, caring for grandchildren is a common and normative experience for many Iranian grandparents. The present study aimed to compare the quality of life of physically active and inactive grandmothers caring and non-caring for grandchildren.

**Methods:**

This analytical cross-sectional study was conducted on 300 grandmothers at the age range of 50 to 70 years old, who lived in Bojnurd, northeastern Iran (2018). Data were collected using the questionnaires of 36-Item Short Form Health Survey (SF-36) and Baecke Physical Activity, and demographic information.

**Results:**

The total scores of quality of life in caring physically active, physically inactive, non-caring physically active and non-caring physically inactive grandmothers were 76.95±6.33, 71.74±9.41, 75.56±5.9, and 56.06±11.23, respectively. There was a significant difference in the comparison of the quality of life score in caring grandmothers in two active and inactive groups (P<0.001). In addition, the quality of life score of non-caring grandmothers indicated a significant difference in physically active and inactive grandmothers (P<0.001).

**Conclusion:**

According to the results of this study, it seems that caring physically active grandmothers have higher quality of life. It can be suggested that the grandmothers who care for their grandchildren may improve their health and quality of life by incorporating the programs to increase physical activities in their daily life.

## Introduction

Grandparents play a major role in caring for children all over the world. It is well recognized that grandparents play a vital economic and social role in providing grandchild care to families. Grandparents caring for grandchildren provide a critical service for both the children and the children's parents. Like other care work, this service has public, as well as private, benefits (1–2). Various factors such as economic value, financial pressures on families and high costs of childcare, working parents and absence of parents due to substance abuse, illness or imprisonment have led to the increasing attention to caring for grandchildren by their grandparents (1, 2–5). It has been shown that in United Kingdom about two third [63%] of grandparents care for their grandchildren (6). Also, about 70% of grandparents in East Asia live with their grandchildren (7). Grandparents play the role of supporters for their grandchildren in critical situations of life and can be considered as a key factor in the relations between parents and children (8). The impact of caring for grandchildren on grandparents' health is particularly associated with the culture of the society and the hours they care for their grandchildren (9). It has been previously shown that caring for grandchildren can led to the sense of satisfaction and improved health in grandparents (5, 10, 11), and their higher quality of life (12) and better cognitive performance (13). Despite some studies showing the positive effects of caring for grandchildren by grandparents' on their quality of life, the results of other studies have indicated that the prevalence of diseases such as depression, diabetes and hypertension is higher in grandmothers who care for their grandchildren and often have more difficulties in performing their daily activities rather than their peers (14). In addition it has been indicated that the grandmothers who are in charge of upbringing their grandchildren, as compared to those who do not have such responsibility or live in families with other generations, are more exposed to caring burden, depression, and anxiety (15). The results of another study showed that stress and activities to care for grandchildren influence grandparents' health (16). Furthermore, caring for little children required more physical activities and the grandmothers with such a responsibility are more likely to suffer from coronary heart disease (17). As the results indicated, physical activities has been considered as one of effective factors on the elderly's quality of life, so that lower physical activity is associated lower quality of life. Following physical activity and health instructions has been identified as positively associated with the improved quality of life in the elderly (18–21). Also, it has been shown that grandparents-grandchildren relationship is significantly contributed to quality of life of older adults (22).

In Iranian cultural background, caring for grandchildren is a common experience of most grandmothers. Considering that the experience of caring for grandchildren deals with challenges that can be different with the experienced investigated in other researches countries, due to different cultural, national, and religious contexts, and since there is no similar study in this regard, the present study aimed at comparing the quality of life in physically active and inactive grandmothers caring and non-caring for their grandchildren.

## Methods

In this analytical cross-sectional study, a total of 300 caring and non-caring grandmothers at the age range of 50 to 70 years old, who live in Bojnurd, northeastern Iran, were evaluated (2018). Seventy five physically active and 75 inactive grandmothers caring for grandchildren and 75 physically active grandmothers and 75 inactive grandmothers non-caring for grandchildren were enrolled in this study and investigated. The participants were selected by random cluster sampling method from different seven areas of the city, according to the areas of expansion unit of health deputy. The main cluster of each center was systematically identified by the number of recorded cases in the integrated health system in each medical health care center. After the main cluster was identified, sampling started by referring to subjects' houses. Then, the sampling was performed from the right-hand side of the household of the cluster of the adjacent households to finding the whole population of each cluster. The samples were selected at the time of referring to houses, according to the inclusion criteria and obtaining the consent forms. Then, the required information was collected by interviews, using research tools. The inclusion criteria were, having at least one grandchild, being at the age range of 50 to 70 years old, having the experience of caring for a grandchild below 6 years old at least in one year ago in the group of caring grandmothers, having physical activity with average intensity over 6 past months in the group of physically active grandmothers, being aware about time, place, and the ability to communicate verbally, not having severe depression and having a normal grandchild. The exclusion criteria were unwillingness to continue participation in the study or failed to answer questionnaires items.

The tools used in this study were the questionnaire of demographic characteristics, the health survey, and Baecke physical activity questionnaire. The researcher-made demographic characteristics questionnaire contains information such as age, education, marital status, housing status, average hour of caring for grandchild, and the age and sex of the grandchild who is cared and the grandmother's relationship with the grandchild (paternal or maternal). The short form 36 (SF-36) health survey is a general tool to measure self-reported health and quality of life, which can be used to measure the health status and health-related quality of life in both healthy and sick populations (23–25). This form has 36 items and designed to measure 8 domains of health status including physical functioning, role physical, bodily pain, general health, social functioning, vitality, mental health, and role emotional. Two general physical and mental components are formed through integration of these subscales. A likert scale (excellent, very good, good, fair, or poor), and yes and no is used to answer different questions of this questionnaire. The maximum score for each domain or subscale is 100 and the minimum score is 0; higher score indicates better quality of life and lower score indicates lower quality of life. The validity of the Persian version of SF-36 was confirmed by Montazeri et al. (24) and Farhadi et al. (26) with correlation coefficients of 0.58 to 0.95, and 0.45 to 0.72, respectively. The reliability of this questionnaire was also confirmed in the study by Montazeri, using internal consistency Cronbach's alpha coefficients as 0.77 to 0.90 (26).

Baecke physical activity questionnaire is a most widely used self-administered tool for measuring a person's habitual physical activities over the past year. This is a 16 item questionnaire, divided across three domains including occupational physical activities, sport, and leisure. Each domain can receive a score from 1 to 5. Within each domains, each question is also given a score from 1 to 5. The first part, i.e. occupational physical activity, has eight questions and the scores are added and divided into eight. The second part, i.e. sport physical activity, contains four questions and the sum of scores is divided into four. The third part is physical activity in leisure time and has four questions and the sum of the scores is divided into four. Finally, the scores of these three parts are added together and the final score shows the person's physical activity. The total score can range between 3 and 15 and higher score indicate higher level of physical activity (27). The validity and reliability of this questionnaire, and its Persian version, has been confirmed in previous studies (27–29).

**Ethical consideration**: The study was carried out in accordance with the Declaration of Helsinki. The present study was approved by the ethics committee of North Khorasan University of Medical Sciences (IR.nkums.REC.1395.34). The researchers explained the objectives of study to the participants, and informed consent was obtained from all participants. Emphasis was placed on the voluntary nature of the participants' involvement in the study. Grandmothers' participation was voluntary and they could end their participation at any time without explanation.

**Sample size estimation:** The estimation of sample size was based on a presumed effect size of 0.4 (30), a statistical power of 95%, and a type I error of 5% using G*Power software, version 3.1.3 with the formula for calculation of sample of correlational studies. The overall proper sample size was found to be 300 participants.

**Statistical analysis**: Data were analyzed using the Statistical Package for the Social Sciences (SPSS) software (version 16.0, SPSS Inc., Chicago, IL, USA) using the descriptive-analytical statistics tests including frequency, mean, analysis of variance, chi square test, independent t-test, and Mann-Whitney U test. Shapiro-Wilk test was used to assess normality of the data. P-value <0.05 was considered statistically significant.

## Results

The Strengthening the Reporting of Observational Studies in Epidemiology (STROBE) checklist was used to report important aspects of this study. In this study, 329 grandmothers were evaluated, of which 300 patients were eligible to be included in this study. Of total grandmothers (n=300) who enrolled in this study, 153 were caring and 147 were non-caring of their grandchildren. All participants had complete data — there was no missing data to address. According to Baecke physical activity questionnaire, 48.36% and 49.65% of caring and non-caring grandmothers were physically inactive. The age mean of the participants was 59.07±5.57 year and 75.57% of grandmothers living with their spouse. Moreover, 46.42% of them had an average income and more than 90% of them had a house. There was a statistically significant difference between physically active and inactive grandmothers in terms of monthly income greater than 10,000,000 Rials (P<0.01). The groups under investigation (caring physically inactive, non-caring physically inactive, caring physically active, non-caring physically active) were homogeneous in terms of the age of grandmothers and the grandchildren's age and sex. The average hours of caring for grandchildren in the physically active group was 23.27±12.72 hours and in the physically inactive group, it was 28.44±19.35 hours in a week (P=0.027). 72.5% of caring grandmothers cared for one grandchild, 21.5% cared for two grandchildren, and 5.95% cared for three grandchildren. In all, 45.5% of total participants were paternal grandmothers and 54.5% were maternal grandmothers (P=0.083).

In terms of total score of quality of life as well as the scores of its physical and mental dimensions in active and inactive grandmothers, there was a statistically significant differences between active and inactive caring grandmothers as well as between active and inactive non-caring grandmothers (p<0.001) (Table 1). In addition, the total scores of quality of life and its physical and mental dimensions had a significant difference in caring grandmothers and non-caring grandmothers (p<0.001) (Table 2).

**Table 1:** Comparison of mental, physical and total scores of quality of life in active and inactive grandmothers

Areas of quality of life	Group of grandmothers	Mean ± SD	*P*-value*
Mental	Active caring grandmother	89.95 ± 8.43	0.006
	Inactive caring grandmother	85.85 ± 10.52	
	Active non-caring grandmother	88.69 ± 7.72	<0.001
	Inactive non-caring grandmother	65.59 ± 13.35	

Physical	Active caring grandmother	63.95 ± 6.25	<0.001
	Inactive caring grandmother	57.63 ± 10.07	
	Active non-caring grandmother	62.42 ± 6.19	<0.001
	Inactive non-caring grandmother	42.51 ± 12.07	

Total score of quality of life	Active caring grandmother	76.95 ± 6.33	<0.001
	Inactive caring grandmother	73.79 ± 9.41	
	Active non-caring grandmother	75.56 ± 5.9	<0.001
	Inactive non-caring grandmother	56.06 ± 11.23	

* Mann–Whitney U test

**Table 2:** Comparison of mental, physical and total scores of quality of life in caring and non-caring grandmothers

Areas of quality of life	Group of grandmothers	Mean ± SD	*P*-value*
Mental	Caring grandmother	87.92 ± 9.62	<0.001
	Non-caring grandmother	79.2 ± 14.47	
Physical	Caring grandmother	60.69 ± 8.99	<0.001
	Non-caring grandmother	52.54 ± 13.81	
Total score of quality of life	Caring grandmother	74.26 ± 8.46	<0.001
	Non-caring grandmother	65.87 ± 13.24	

* Mann–Whitney U test

Regarding bodily pain, there was no significant difference between caring physically active and inactive grandmothers (P=0.107) and also between non-caring physically active and inactive grandmothers (P=0.7). In addition, the domain of role emotional (P=0.055) and mental health (P=0.079) was not significantly different between active and inactive caring grandmothers (Table 3). In comparison of all domains of quality of life in caring and non-caring grandmothers, except in the domain of bodily pain that was not significantly different between caring grandmothers and non-caring grandmothers (p=0.991), other domains had a significant difference differences (p<0.001) (Table 4).

**Table 3:** Comparison of scores of different areas of quality of life of in active and inactive grandmothers

Areas of quality of life	Group of grandmothers	Mean ± SD	*P*-value
Social functioning	Active caring grandmothers	94.42 ± 14.79	0.006
	Inactive caring grandmothers	89.08 ± 16.3	
	Active non-caring grandmothers	93.24 ± 13.43	<0.001
	Inactive non-caring grandmothers	74.31 ± 21.63	
Vitality	Active caring grandmothers	83.04 ± 8.51	0.001
	Inactive caring grandmothers	77.15 ± 12.8	
	Active non-caring grandmothers	81.82 ± 9.56	<0.001
	Inactive non-caring grandmothers	61.57 ± 14.16	
Mental health	Active caring grandmothers	86.44 ± 9.22	0.07
	Inactive caring grandmothers	82.68 ± 13.22	
	Active non-caring grandmothers	84.64 ± 8.34	<0.001
	Inactive non-caring grandmothers	69.2 ± 12.8	
Role emotional	Active caring grandmothers	95.72 ± 10.7	0.05
	Inactive caring grandmothers	95.14 ± 10.38	
	Active non-caring grandmothers	95.04 ± 11.61	0.001
	Inactive non-caring grandmothers	73.28 ± 20.36	
General health	Active caring grandmothers	71.95 ± 10.28	0.005
	Inactive caring grandmothers	65.45 ± 14.63	
	Active non-caring grandmothers	70.49 ± 11.05	0.001
	Inactive non-caring grandmothers	49.65 ± 15.36	
Bodily pain	Active caring grandmothers	42.39 ± 9.49	0.10
	Inactive caring grandmothers	39.18 ± 10.97	
	Active non-caring grandmothers	41.08 ± 11.3	0.70
	Inactive non-caring grandmothers	40.96 ± 13.21	
Role physical	Active caring grandmothers	47.12 ± 7.32	0.01
	Inactive caring grandmothers	42.88 ± 12.12	
	Active non-caring grandmothers	46.45 ± 9.56	<0.001
	Inactive non-caring grandmothers	21.06 ± 18.14	
Physical functioning	Active caring grandmothers	94.32 ± 12.17	<0.001
	Inactive caring grandmothers	83.03 ± 19	
	Active non-caring grandmothers	91.68 ± 12.55	<0.001
	Inactive non-caring grandmothers	58.38 ± 23.1	

* Mann–Whitney U test

**Table 4:** Comparison of scores of different areas of quality of life of in caring and non-caring grandmothers

Areas of quality of life	Group of grandmothers	Mean ± SD	*P*-value
Social Functioning	Caring grandmothers	91.67 ± 15.77	<0.001
	Non-caring grandmothers	83.84 ± 20.28	
Vitality	Caring grandmothers	80 ± 11.29	<0.001
	Non-caring grandmothers	71.76 ± 15.74	
Mental Health	Caring grandmothers	84.6 ± 11.5	<0.001
	Non-caring grandmothers	76.97 ± 13.25	
Role Emotional	Caring grandmothers	95.42 ± 10.51	<0.001
	Non-caring grandmothers	84.24 ± 19.78	
Bodily Pain	Caring grandmothers	40.73 ± 10.37	0.99
	Non-caring grandmothers	41.02 ± 12.24	
Role Physical	Caring grandmothers	44.39 ± 10.28	<0.001
	Non-caring grandmothers	33.84 ± 18.69	
Physical functioning	Caring grandmothers	88.49 ± 16.98	<0.001
	Non-caring grandmothers	75.15 ± 24.92	
General Health	Caring grandmothers	68.6 ± 13.08	<0.001
	Non-caring grandmothers	60.14 ± 16.93	

*Mann–Whitney U test

## Discussion

The results of the present study aimed at investigating the quality of life in active and inactive grandmothers caring and non-caring for their grandchildren showed that the quality of life in grandmothers who cared for their grandchildren and those who were physically active was higher than non-caring and physically inactive grandmothers. The scores of mental and physical dimensions and the total score of quality of life in caring grandmothers and active ones were higher than non-caring and inactive grandmothers. Physically active grandmothers who cared for their grandchildren had the highest score of quality of life among other grandmothers.

In a study by Lu and Liu, conducted by a similar tool, it was indicated that there was no significant difference in the quality of life between caring and non-caring older grandparent (31). Possible explanations for this inconsistent finding may be a small sample size in this study and also cultural differences in the societies under investigation (5). In the present study, the domain of role physical and bodily pain had the lowest score. In a study by Lo and Liu, like the finding of this study, the domain of role physical had the lowest score, while contrary the result of this study, the domain of bodily pain had the highest score (31). These differences may be due to cultural differences and their effects on emotional or behavioral reactions to pain. In this study, like the present study, it was found that physical component had lower score than mental component, which can related it to the old age and the physical problem that emerge with getting old.

The caring grandmothers in this study had higher physical and mental health than the non-caring ones. The results of some studies showed a positive relationship between caring for grandchildren and better self-rated health (2, 9, 10, 17, 32–34).

It has been previously shown that caring for grandchildren reduces time for self-care, such as exercising and going to the doctor, and time for engaging in hobbies and socializing. The stress of care-giving may cause or exacerbate poor health behaviors, such as smoking. Also, care-giving grandparents may reduce hours of paid employment, which may lead to financial distress (34). In the present study, the average hour to care for grandchildren was 25 hours a week, which had no negative effect on grandmother's health. Caring grandmothers had a better level of physical and mental health than non-caring grandmothers. The same results were found in a study conducted in Europe. It indicated that longer care (daily or at least 15 hours a week) and limited care did not have any negative effect on grandparents' health, while it was beneficial to their mental and role physical and was associated with better self-report of health. Moreover, there was no significant relationship between caring for grandchildren and depression symptoms or inability in doing daily activities (32).

Another study conducted in Europe had consistent results with the present study; it showed that caring grandparents had higher quality of life as compared to non-caring grandparents (12). Also, in a study in Taiwan it has been shown that compared with non-caregivers, caring grandparents were more likely to report better self-rated health, higher life satisfaction, and fewer depressive symptoms (35). Another study in China indicated that grandparents who cared for grandchildren had better mental and physical health, compared with non-caregivers (36). Despite the similar results with those found in European studies, studies in America indicated a negative relationship between caring for grandchildren and caring grandparents' health. Especially, the grandparents who regularly care for their grandchildren or live with them or care for them 15 hours a week or more were more likely to have health problems and depression symptoms rather than those who care for their grandchildren less hours a week or did not care for them (9, 14, 34).

Unlike the results of the present study in that caring for grandchildren was associated with higher scores in mental dimension, some studies in America showed the relationship between depression and caring for grandchildren (15, 37). It has been shown that grandparents who care for their grandchildren without their parents were more likely to experience negative changes in health behaviors and depression. However, the grandmothers who started or continued daily care for their grandchildren had better health status than non-caring grandmothers (33, 34). The results of a study in Thailand revealed that regularly taking care of young grandchildren by grandparents have a negative impact on self-rated health, functional limitations and psychological well-being (38).

The results of this study showed that there was a significant difference in the quality of life between active caring and inactive caring grandmothers, as well as between active non-caring and inactive non-caring grandmothers. Grandmothers who were physically active obtained higher score of quality of life than those who were physically inactive. Similar finding was found in various studies in Iran; the mean score of quality of life in older adults who were physically active was significantly higher than that of physically inactive elderlies (39, 40). Furthermore, many studies from different parts of the world found a strong relationship between grater level of physical activity and higher quality of life (18, 41, 42). According to the results, there was no significant difference in the domains of mental health and role emotional between the active and inactive caring grandmothers, however there was a significant difference between active and inactive non-caring grandmothers. Caring for grandchildren by both physically active and inactive grandmothers can be associated with an increased in their quality of life in both groups, which can justify this lack of difference of quality of life’ scores between physically active and inactive grandmothers. According to the results of this study, the effect of physical component of quality of life was significant. These results are consistent with the results of some previous studies (43, 44). However, Hamedinia et al. found no significant difference in the physical problems between active subjects and inactive subjects (45). Considering the fact that physical activity is one of the most important factors that can contribute to losing weight in long time and reducing the risk of cardiovascular disease, arterial diseases, and type 2 diabetes (20, 21), it can be expected that active people have less physical problems.

In the domain of mental health in this study, like some previous studies, physically active grandmothers in both caring and non-caring groups had higher mental health (43). The results of other studies, on the other hand, were not consistent with the results of this study (44, 45). This difference may be due to difference conditions of working environment, social class, income, and geographical environment (45). In this study, like many other studies, active grandmothers had significantly less physical restrictions than inactive grandmothers. They also had higher vitality and general health (43–45). Since exercise boosts muscular strength and endurance, cardiovascular endurance, body speed and balance, and makes daily activities easier and makes the feeling of pleasure, joy, and calmness by increasing the secretion of morphine-like neurotransmitters such as endorphins and serotonin in blood, this result is not unexpected. Unlike some studies (45), the score of social functioning in physically active grandmothers in this study was higher than that of inactive one. Similar finding was found in other studies (43, 44). Participation in sport activities can help to improve social relations, friendship, and normal relationship with peers, which can be one of the reasons for obtaining high scores in the variable of social functioning of physically active rather than physically inactive grandmothers.

In this study, it was out of the researcher's control to precisely monitor the variables, which is considered a limitation of the study. Uncertainties in answering questions, low educational level, and in some cases, the grandmothers' hearing and physical impairment are considered as limitation of this study, which were partially resolved by data collecting through interviews and, if possible, in several sessions. A number of limitations in the present study deserve to be mentioned. First, the cross-sectional design of the current study limits our ability to form firm conclusions regarding causality. Second, in the present study only grandmothers at the age range of 50 to 70 years old, who lived in Bojnurd, northeastern Iran were evaluated. Therefore our finding may not be generalizable to all grandmothers in the country or grandmothers in other countries with possible different ethnicity, religions, cultures, and political backgrounds. In conclusion, according to the results of this study it seems that the physically active grandmothers and the grandmothers who cared for their grandchildren had higher quality of life. In fact, daily care for grandchildren is associated with grandmothers' increased physical activity. Therefore, it can has the same positive effect as sport activities on grandmothers' physical functioning. Moreover, it seems that spending time with grandchildren can make grandmothers happy and has positive effect on their spirit, and hence, the grandmothers can be encouraged to care for their grandmothers and doing physical activities to improve their quality of life.

## References

[1] Burn K, Szoeke C (2016). Boomerang families and failure-to-launch: Commentary on adult children living at home. Maturitas.

[2] Baker LA, Silverstein M, Putney NM (2008). Grandparents raising grandchildren in the United States: Changing family forms, stagnant social policies. J Soc Soc Policy.

[3] Bagchegi O, Tabatabaeichehr M, Lashkardoost H, Mortazavi H (2021). The Effect of Education Based on Kolb's Learning Style on Selfcare Behaviors of the Elderly with Type II Diabetes: A Randomized, Clinical Trial. Ethiop J Health Sci.

[4] Fuller-Thomson E, Minkler M (2001). American grandparents providing extensive child care to their grandchildren: Prevalence and profile. Gerontologist.

[5] Goodman C, Silverstein M (2002). Grandmothers raising grandchildren: Family structure and well-being in culturally diverse families. Gerontologist.

[6] Fergusson E, Maughan B, Golding J (2008). Which children receive grandparental care and what effect does it have?. J Child Psychol Psychiatry.

[7] Yasuda T, Iwai N, Chin-Chun Y, Guihua X (2011). Intergenerational coresidence in China, Japan, South Korea and Taiwan: comparative analyses based on the East Asian social survey 2006. J Comp Fam Stud.

[8] Silva DMd, Vilela ABA, Nery AA, Duarte ACS, Alves MdR, Meira SS (2015). Dynamics of intergenerational family relationships from the viewpoint of elderly residents in the city of Jequié (Bahia), Brazil. Cien Saude Colet.

[9] Chen F, Liu G (2012). The health implications of grandparents caring for grandchildren in China. J Gerontol B Psychol Sci Soc Sci.

[10] Grundy EM, Albala C, Allen E, Dangour AD, Elbourne D, Uauy R (2012). Grandparenting and psychosocial health among older Chileans: A longitudinal analysis. Aging Ment Health.

[11] Arpino B, Bordone V, Balbo N (2018). Grandparenting, education and subjective well-being of older Europeans. Eur J Ageing.

[12] Neuberger FS, Haberkern K (2014). Structured ambivalence in grandchild care and the quality of life among European grandparents. Eur J Ageing.

[13] Sneed RS, Schulz R (2019). Grandparent caregiving, race, and cognitive functioning in a population-based sample of older adults. J Aging Health.

[14] Minkler M, Fuller-Thomson E (1999). The health of grandparents raising grandchildren: results of a national study. Am J Public Health.

[15] Musil CM, Warner CB, Zauszniewski JA, Jeanblanc AB, Kercher K (2006). Grandmothers, caregiving, and family functioning. J Gerontol B Psychol Sci Soc Sci.

[16] Grinstead LN, Leder S, Jensen S, Bond L (2003). Review of research on the health of caregiving grandparents. J Adv Nurs.

[17] Ku L-JE, Stearns SC, Van Houtven CH, Lee S-YD, Dilworth-Anderson P, Konrad TR (2013). Impact of caring for grandchildren on the health of grandparents in Taiwan. J Gerontol B Psychol Sci Soc Sci.

[18] Cho K-O (2014). Sleep duration and self-rated health are independently associated with physical activity level in the Korean population. Iranian J Publ Health.

[19] Wen CP, Wai JPM, Tsai MK, Yang YC, Cheng TYD, Lee M-C (2011). Minimum amount of physical activity for reduced mortality and extended life expectancy: a prospective cohort study. Lancet.

[20] Cho KO, Nam SN, Kim YS (2011). Assessments of nutrient intake and metabolic profiles in Korean adolescents according to exercise regularity using data from the 2008 Korean National Health and Nutrition Examination Survey. Nutr Res Pract.

[21] Kim YS, Park YS, Allegrante JP, Marks R, Ok H, Cho KO (2012). Relationship between physical activity and general mental health. Prev Med.

[22] Momtaz YA, Vidouje MM, Foroughan M, Sahaf R, Laripour R (2018). Grandparents - Grandchildren Relationship in Iran, 2017. Clin Pract Epidemiol Ment Health.

[23] Asghari Moghaddam M, Faghehi S (2003). Validity of the SF-36 Health Survey Questionnaire in Two Iranian Samples. J Clin Psychol Pers.

[24] Montazeri A, Goshtasebi A, Vahdaninia M, Gandek B (2005). The Short Form Health Survey (SF-36): translation and validation study of the Iranian version. Qual Life Res.

[25] Lins L, Carvalho FM (2016). SF-36 total score as a single measure of health-related quality of life: Scoping review. SAGE Open Med.

[26] Farhadi A, Foroughan M, Mohammadi F (2011). The quality of life among rural elderlies a cross-sectional study. Iran J Ageing.

[27] Baecke JA, Burema J, Frijters JE (1982). A short questionnaire for the measurement of habitual physical activity in epidemiological studies. Am J Clin Nutr.

[28] Sanaei M, Zardoshtian S, Noruzi SHR (2013). The effect of physical activities on quality of life and hope life in older adults of Mazandaran province. Sport Manage Rev.

[29] Sadeghisani M, Dehghan Manshadi F, Azimi H, Montazeri A (2016). Validity and Reliability of the Persian Version of Baecke Habitual Physical Activity Questionnaire in Healthy Subjects. Asian J Sports Med.

[30] Neely-Barnes SL, Carolyn Graff J, Washington G (2010). The health-related quality of life of custodial grandparents. Health Soc Work.

[31] Lo M, Liu YH (2009). Quality of life among older grandparent caregivers: a pilot study. J Adv Nurs.

[32] Di Gessa G, Glaser K, Tinker A (2016). The impact of caring for grandchildren on the health of grandparents in Europe: A lifecourse approach. Soc Sci Med.

[33] Ko P-C, Hank K (2014). Grandparents caring for grandchildren in China and Korea: Findings from CHARLS and KLoSA. J Gerontol B Psychol Sci Soc Sci.

[34] Hughes ME, Waite LJ, LaPierre TA, Luo Y (2007). All in the family: The impact of caring for grandchildren on grandparents' health. J Gerontol B Psychol Sci Soc Sci.

[35] Ku LJ, Stearns SC, Van Houtven CH, Lee SY, Dilworth-Anderson P, Konrad TR (2013). Impact of caring for grandchildren on the health of grandparents in Taiwan. J Gerontol B Psychol Sci Soc Sci.

[36] Xu H (2019). Physical and mental health of Chinese grandparents caring for grandchildren and great-grandparents. Soc Sci Med.

[37] Bachman HJ, Chase-Lansdale PL (2005). Custodial grandmothers' physical, mental, and economic well-being: Comparisons of primary caregivers from low-income neighborhoods. Fam Relat.

[38] Komonpaisarn T, Loichinger E (2019). Providing regular care for grandchildren in Thailand: An analysis of the impact on grandparents' health. Soc Sci Med.

[39] Hamidizadeh S, Ahmadi F, Aslani Y, Etemadifar S, Salehi K, Kordeyazdi R (2007). Study effect of a group-based exercise program on the quality of life in older men and women in 2006–2007. J Shaheed Sadoughi Univ Med Sci.

[40] Bazrafshan MRe, Hoseini MA, Rahgozar M, Madah BS (2007). The Effect of Exercise in Elderly Women's Quality of Life. Iran J Ageing.

[41] Halaweh H, Willen C, Grimby-Ekman A, Svantesson U (2015). Physical activity and health-related quality of life among community dwelling elderly. J Clin Med Res.

[42] Raggi A, Corso B, Minicuci N, Quintas R, Sattin D, De Torres L (2016). Determinants of quality of life in ageing populations: Results from a cross-sectional study in Finland, Poland and Spain. PLoS One.

[43] Hasanpour-Dehkordi A, Rastar A (2016). Effect of progressive muscle relaxation on social performance and quality of life in ageing. Iran J Ageing.

[44] Sori A, Shabani Moghadam K, Soury R (2016). The effect of physical activity on quality of life in elder women in Kermanshah province. Appl Res Sport Manag.

[45] Hamedinia M, Golestani A (2004). Health-related quality of life in physically active and sedentary lecturers in Sabzevar universities. Olympic.

